# Supplementary material for: Detecting translational regulation by change point analysis of ribosome profiling data sets
Source: RNA. 2014 Oct;20(10):1507–18. doi: 10.1261/rna.045286.114 (PMC4174433; doi:10.1261/rna.045286.114)
Supplement: Supplemental Material [file supp_20_10_1507__index.html]

Detecting translational regulation by change point analysis of ribosome profiling data sets — Detecting translational regulation by change point analysis of ribosome profiling data sets — Supplemental Material 

# Detecting translational regulation by change point analysis of ribosome profiling data sets

## Supplemental Material

**Files in this Data Supplement:**

- Supp Material.pdf
- Supp Table S2.xlsx
- Supp Materials.zip
