# Supplementary material for: Detecting translational regulation by change point analysis of ribosome profiling data sets
Source: RNA. 2014 Oct;20(10):1507–18. doi: 10.1261/rna.045286.114 (PMC4174433; doi:10.1261/rna.045286.114)
Supplement: Supplemental Material [file supp_045286.114_SuppMaterial.pdf]

Figure S1

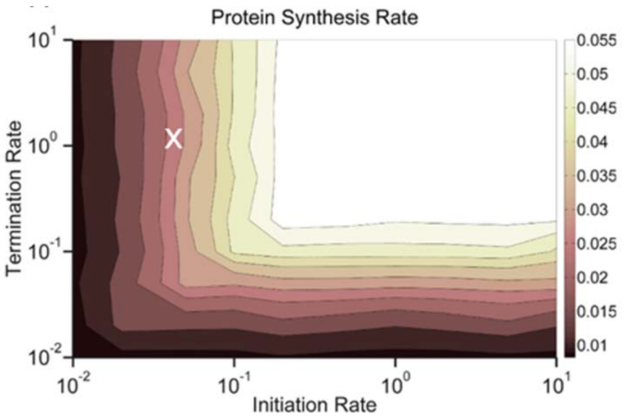

Figure S1. TASEP simulations of protein synthesis. Protein synthesis rate for different initiation and termination rates, (x) marks the average initiation and termination rates for eukaryotes. Unless stated otherwise, $k_{Ei} = 1$, $k_I = 0.1$, $k_T = 1$, $k_{Alt} = 0$, $k_{Di} = 0$. For all simulations, $N = 500$, $L = 10$, $n = 100000$ (number of samples taken from the simulation).

Figure S2

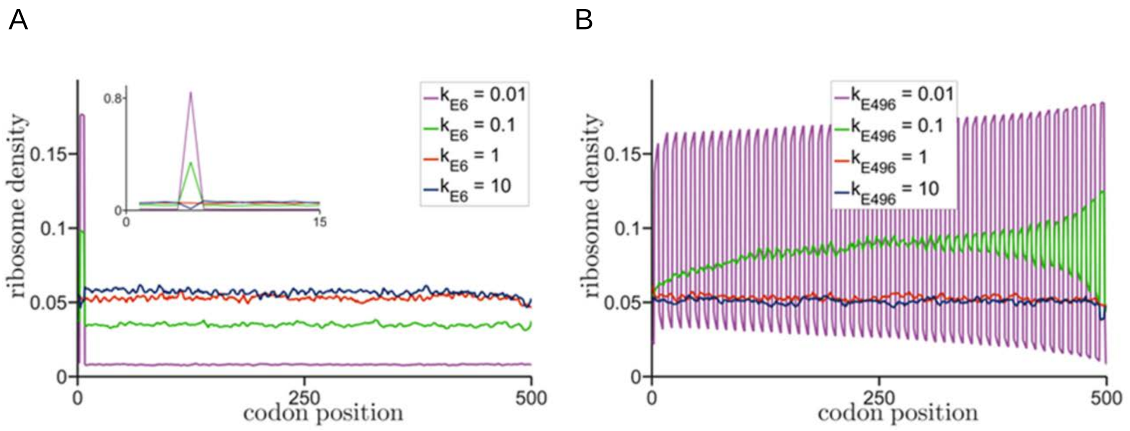

Figure S2. Ribosome profiles for slow elongation. Ribosome density **(**A) for slow elongation at 5' end of CDS and (B) at 3' end of CDS. Unless stated otherwise, $k_{Ei} = 1$, $k_I = 0.1$, $k_T = 1$, $k_{Alt} = 0$, $k_{Di} = 0$. $N = 500$, $L = 10$, $n = 100000$ (number of samples taken from the simulation).

Figure S3

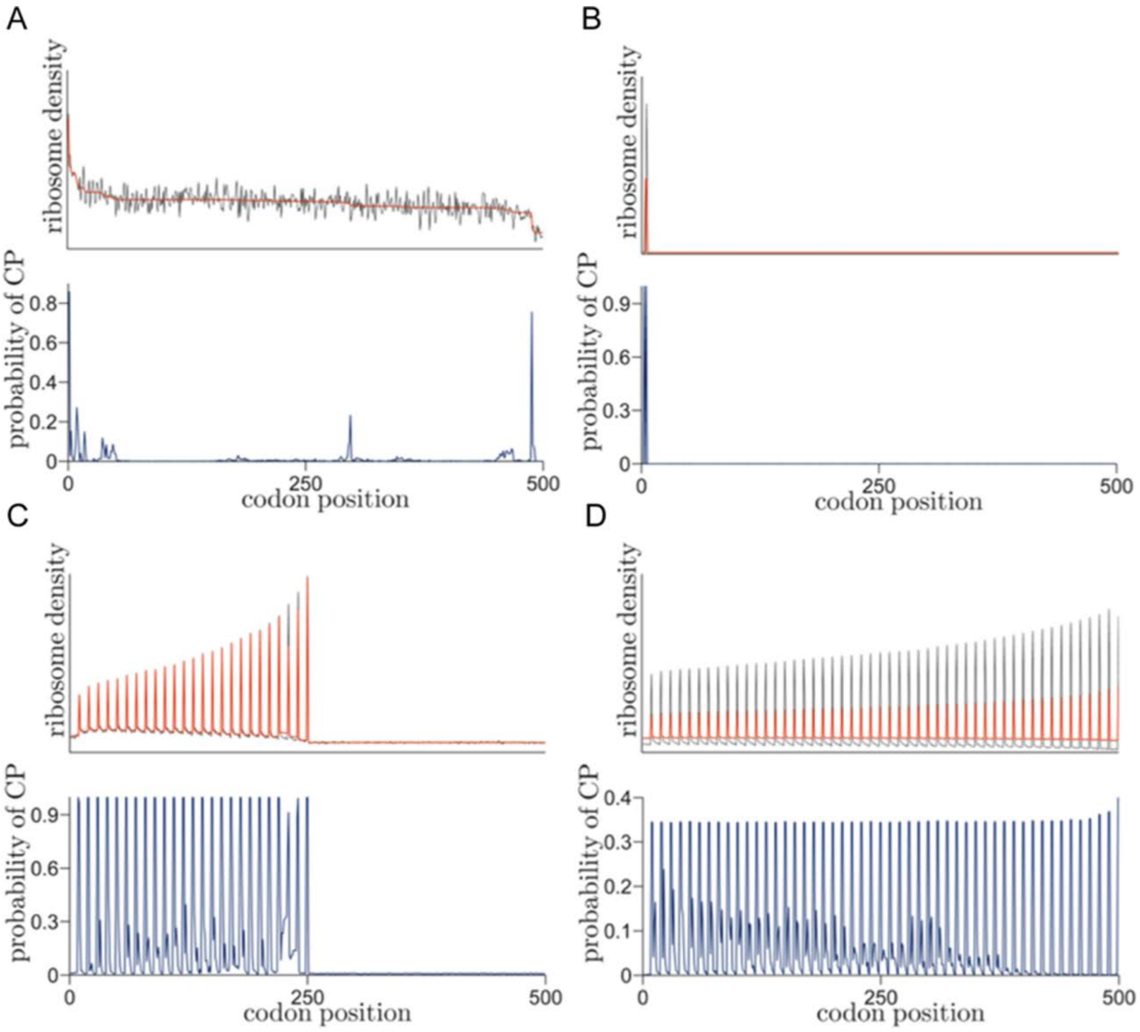

A
B
C
D
ribosome density
probability of CP
0.8
0.6
0.4
0.2
0
0.9
0.3
0.4
0.1
250
500
codon position

Figure S3 (continued)

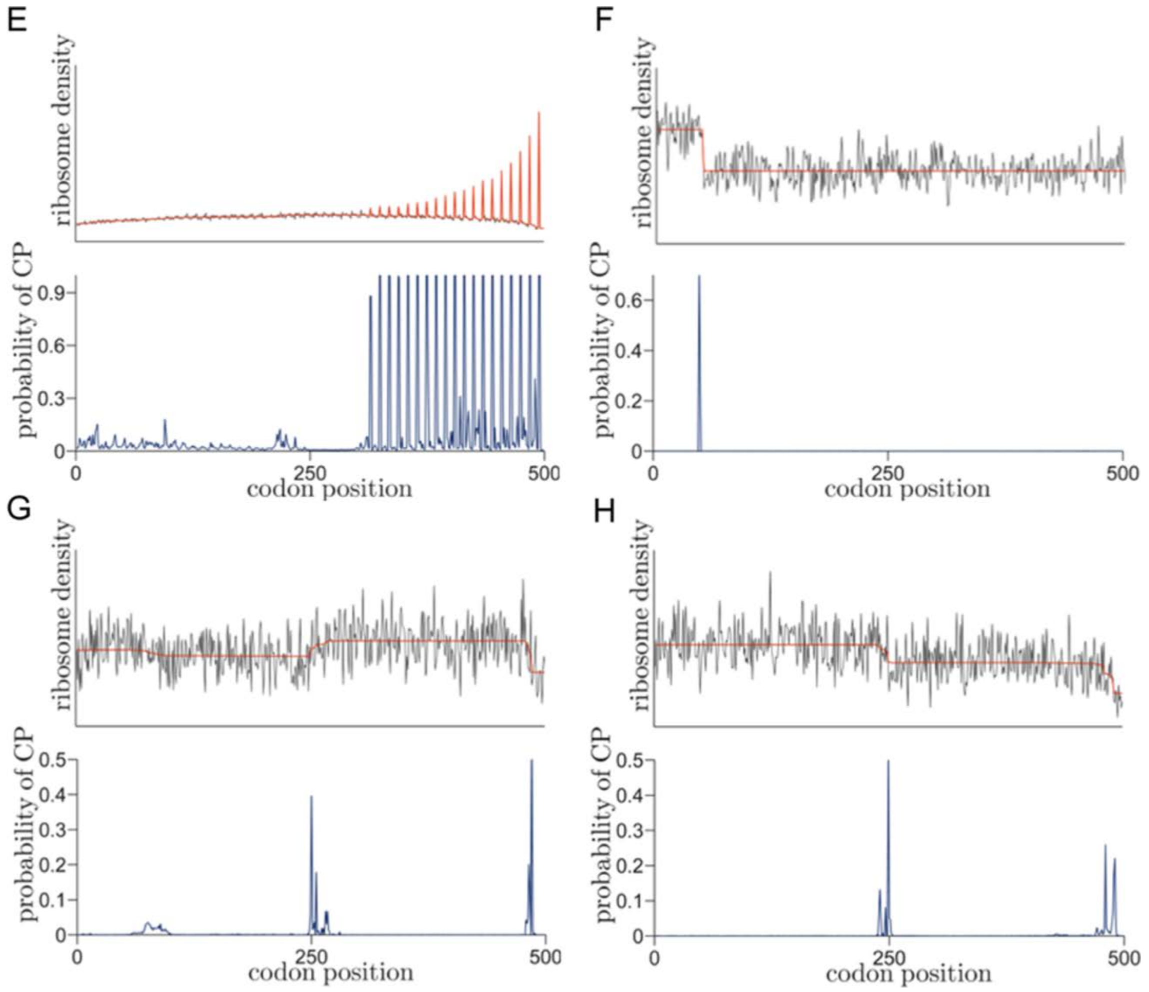

Figure S3. Change point detection for computationally generated ribosome profiles. (A) Estimate of ribosome density (top) and probability of CP (bottom) at high initiation rates – $k_I = 1$, (B) slow codons at the beginning - $k_{E6} = 0.01$, (C) middle - $k_{E251} = 0.01$, and D) end of the CDS - $k_{E496} = 0.1$, E) low termination rates - $k_T = 0.01$, F) ramp caused by slow elongation at 5' of CDS – $k_{E1\text{-}30} = 0.85$. G) alternative initiation – $k_{Alt} = 0.01$ and H) ribosome drop-off/alternative termination – $k_D = 0.01$. Unless stated otherwise, $k_{Ei} = 1$, $k_I = 0.1$, $k_T = 1$, $k_{Alt} = 0$, $k_{Di} = 0$.

Figure S4

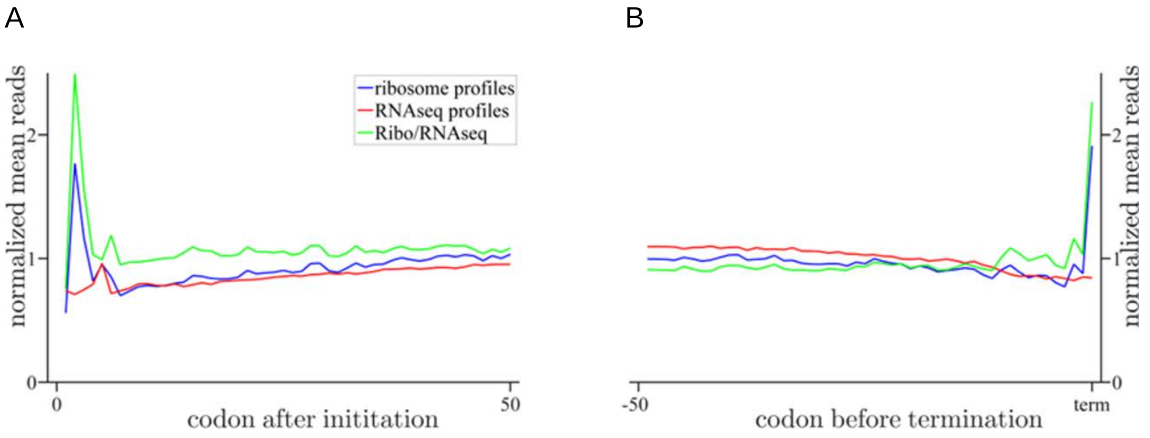

Figure S4. Average ribosome, RNA-Seq and normalized profiles. Average read densities for Ribo-Seq, RNA-Seq and Ribo/RNA are shown for 8,933 well-expressed mRNAs aligned at (A) the start codon and (B) the stop codon. All read densities are normalized against the mean number of reads of the profiles and against their length.

Figure S5

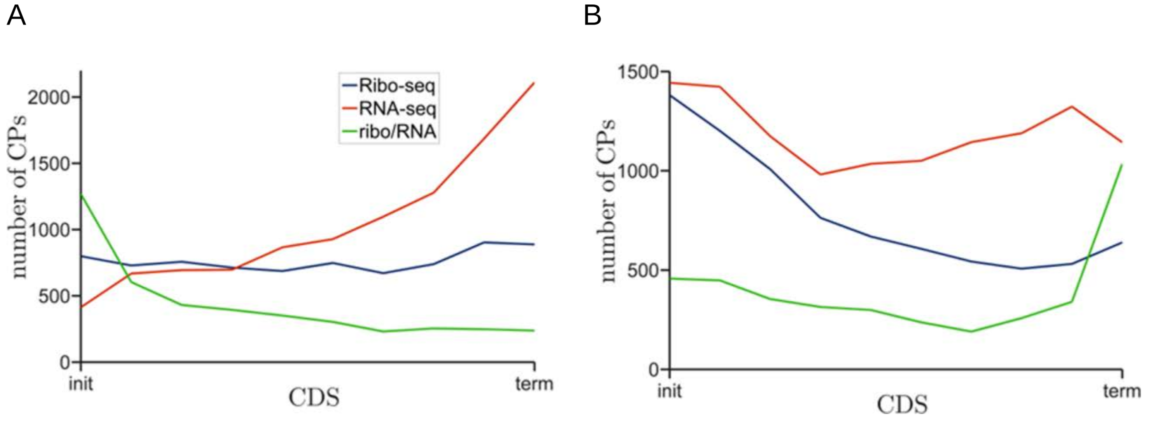

Figure S5. Position of CPs found in ribosome, RNA-Seq and ribo/RNA profiles. A) CPs of decreases in RNA read density. B) CPs of increases in RNA read density.

Figure S6

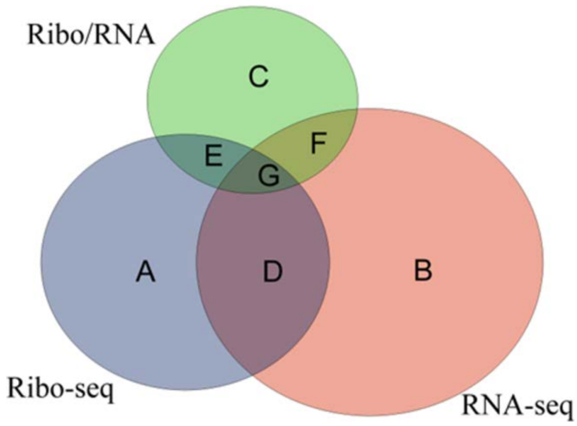

Figure S6. Change points found in ribosome, RNA-Seq and Ribo/RNA profiles. CPs found in Ribo-Seq only (A = 7185), RNA-Seq only (B = 13740), ribo/RNA only (C = 3962), Ribo-Seq and RNA-Seq (D = 5664), Ribo-Seq and ribo/RNA (E = 1355), RNA-Seq and ribo/RNA (F = 1659) and all three (G = 1279). CPs found in different profiles were considered to be the same CP if their 95 % confidence intervals overlapped (see Methods).

Figure S7

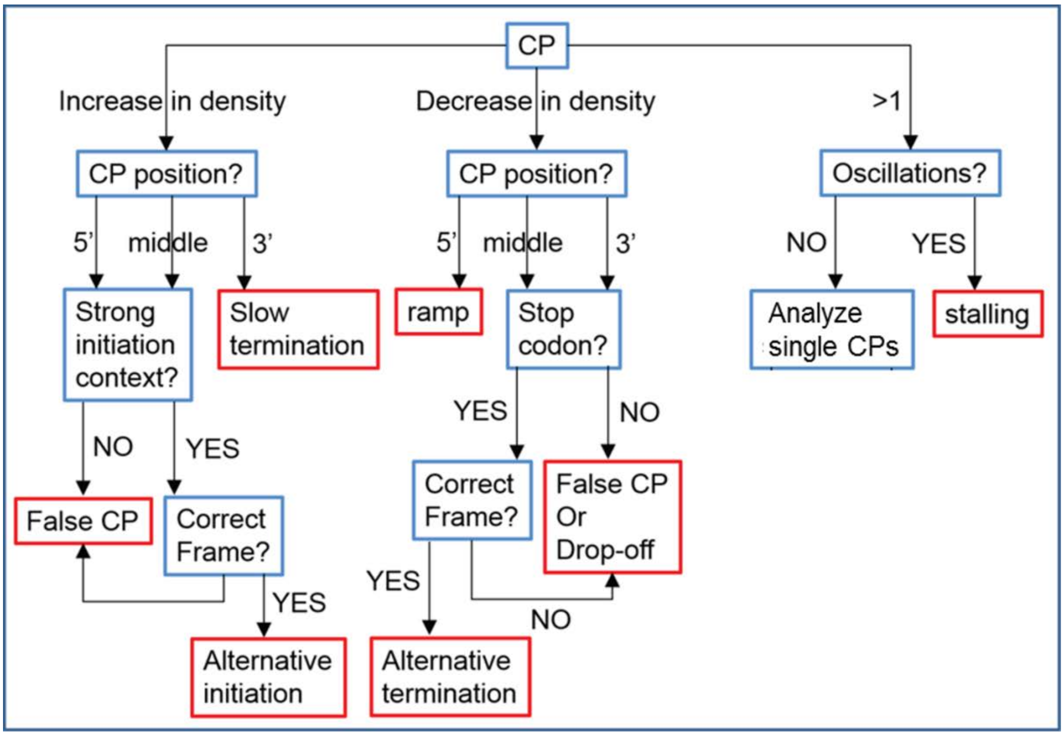

Figure S7. Decision tree for classification of CPs. The decision tree is based on the following criteria: 1) whether the CP analysis detected an increase or decrease in signal density; 2) the position of the CP on the CDS (CPs close to 5' end of the CDS are more likely to represent alternative initiation, while CPs at the 3' end alternative termination); 3) presence of a premature stop codon in any translation frame in proximity of a CP; 4) presence of initiation sequence of nucleotides in any translation frame in proximity of a CP and 5) evidence of a translational frameshift before the CP. After automatically classifying the CPs, the classification was confirmed (or refuted) by visually comparing the profiles to the genomic information available in UCSC Genome Browser.

Figure S8

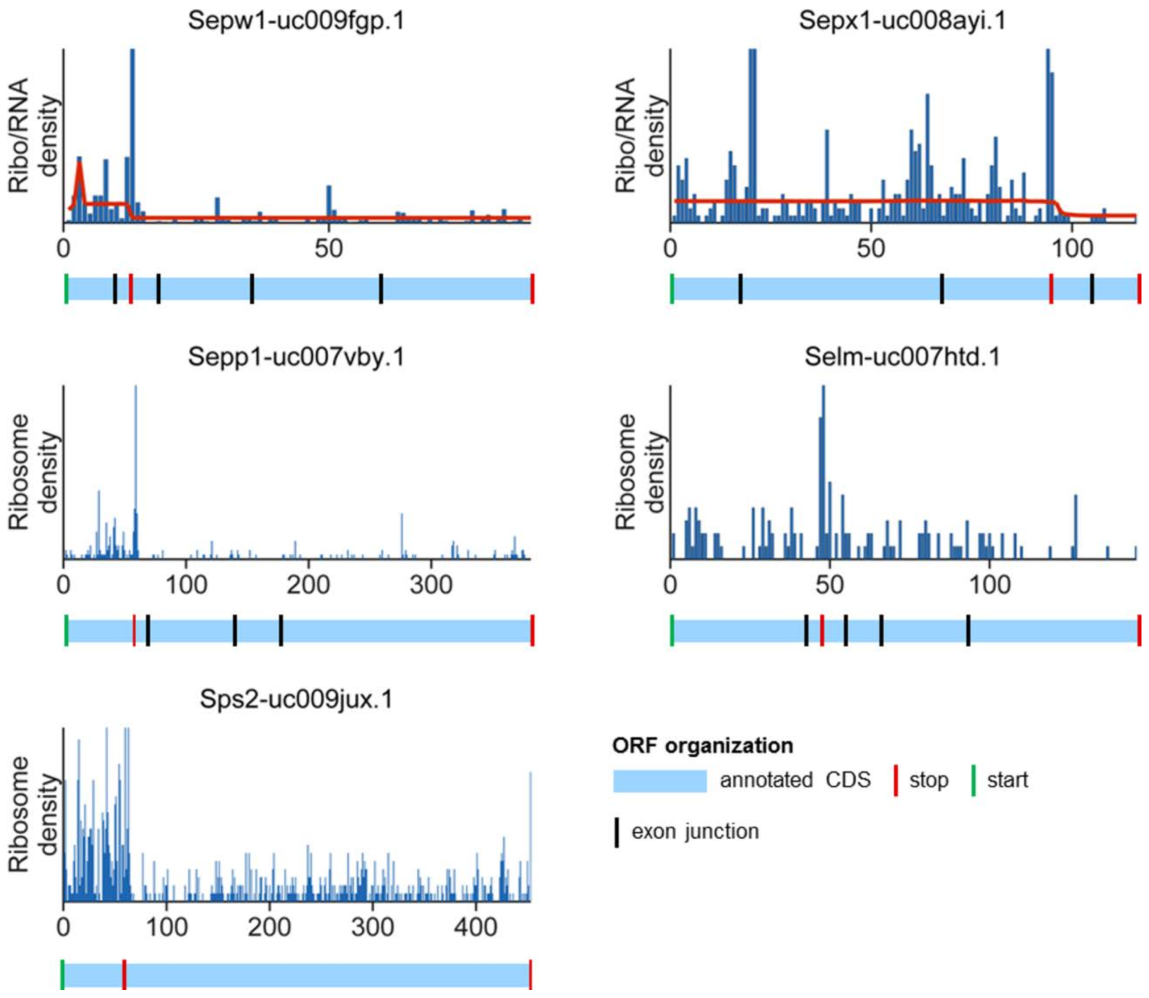

Figure S8. Other selenoprotein genes with alternative termination: *Sepw1*, *Sepx1*, *Sepp1*, *Selm*, *Sps2*. Ribo/RNA or ribosome profiles are shown in blue and for *Sepw1* and *Sepx1*, the segments of equal density estimated by the change point algorithm are shown in red. The annotated CDSs are shown below the profiles plots in light blue. Green vertical lines indicate annotated start codons, red lines indicate annotated stop codons and/or the first stop codon encountered by the ribosome, and black lines indicate exon-exon junctions.

Figure S9

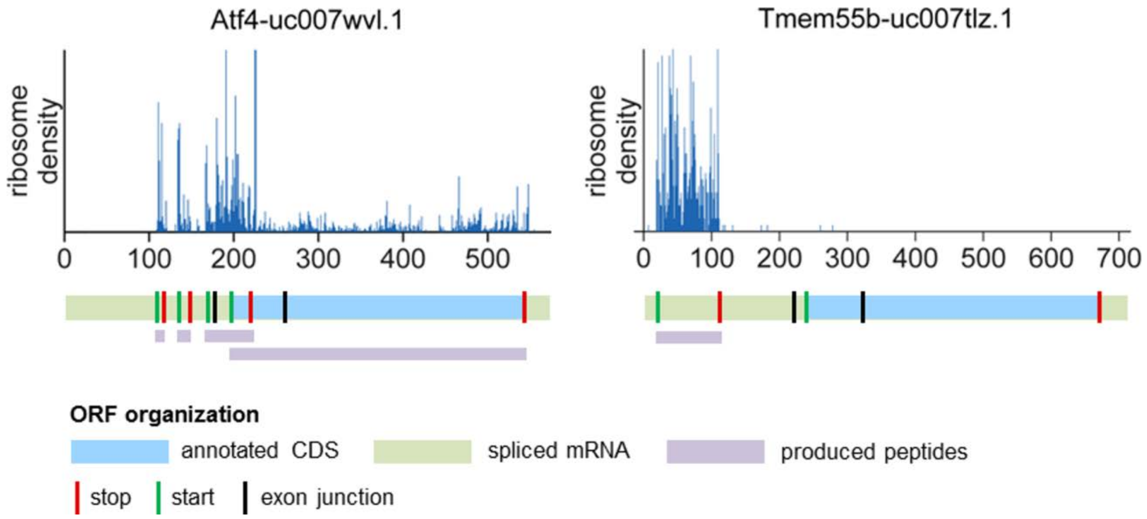

Figure S9. Alternative termination after 5'UTR alternative initiation. Atf4 and Tmem55b are both translated in an alternative reading frame (Atf4 in frame -1, Tmem55b in frame +1), with the alternative initiation codon located in the 5'UTR and the termination in the middle of the annotated CDS. The mRNA is shown below the profiles in light green, with the annotated CDS in light blue synthesised peptide in light purple. Green vertical lines indicate the annotated start codons and putative alternative start codons, red lines indicate annotated stop codons and/or the first stop codon encountered by the ribosome after initiation, and black lines indicate exon-exon junctions.

Figure S10

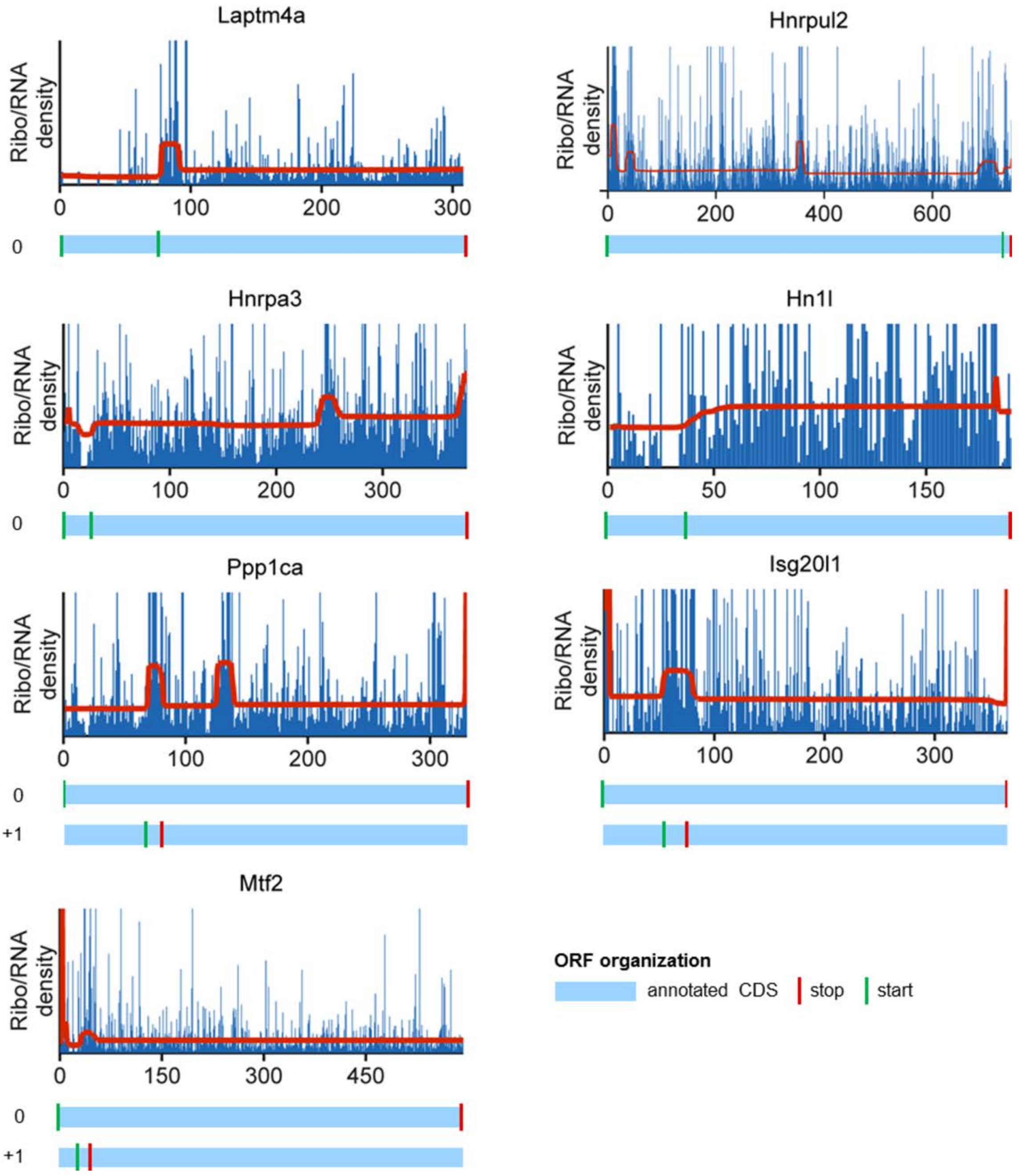

Laptm4a
Hnrpul2
Hnrpa3
Hn1l
Ppp1ca
Isg20l1
Mtf2
Ribo/RNA density
0
100
200
300
400
600
50
150
450
+1
ORF organization
annotated CDS
stop
start

Figure S10 continued

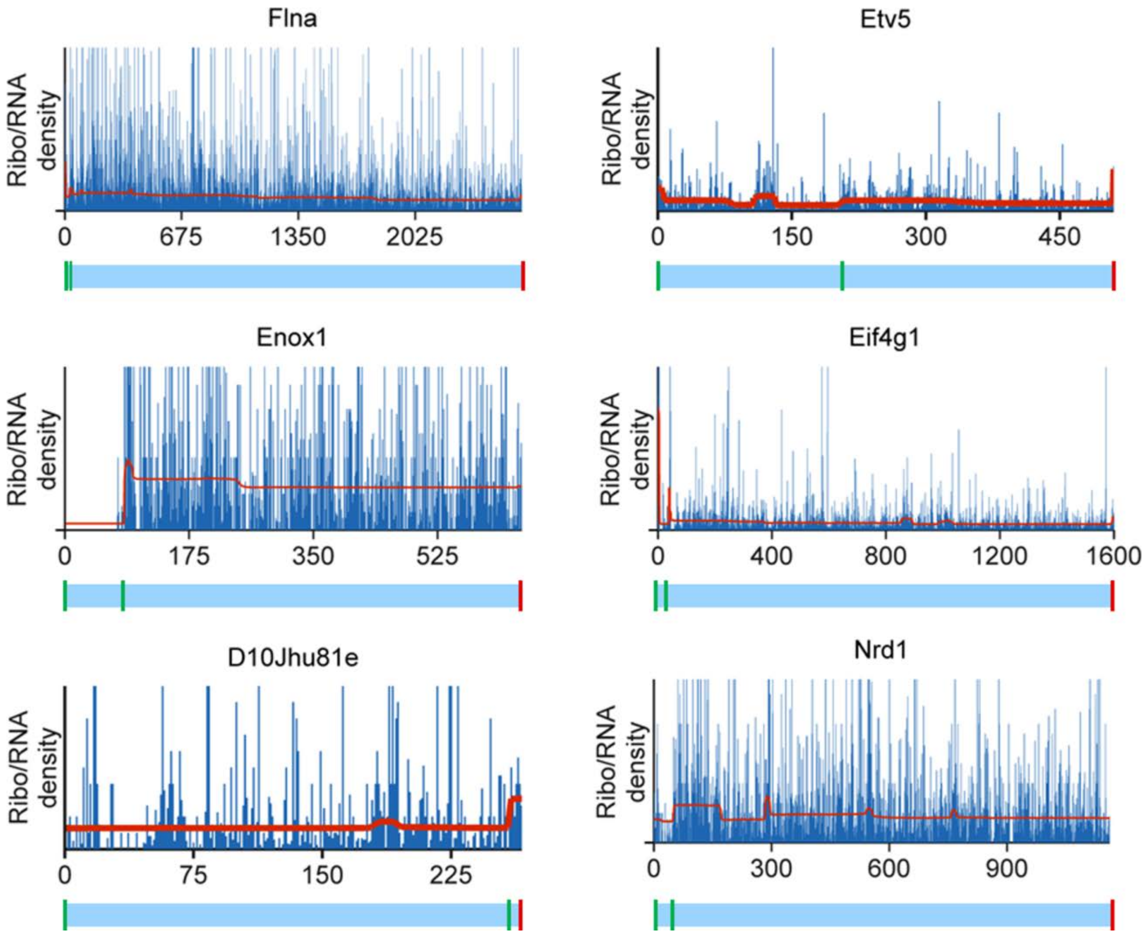

Figure S10. Genes with alternative initiation found after comparing CPs with alternative initiation sites discovered by (Ingolia et al. 2011). Ribo/RNA profiles are shown in blue and the segments of equal density estimated by the change point algorithm are shown in red. The annotated CDSs are shown below the profiles plots in light blue. Green vertical lines indicate annotated start codons and the newly discovered alternative start codons, while red lines indicate the annotated stop codons or the first stop codon encountered by the ribosome after initiation.

Figure S11

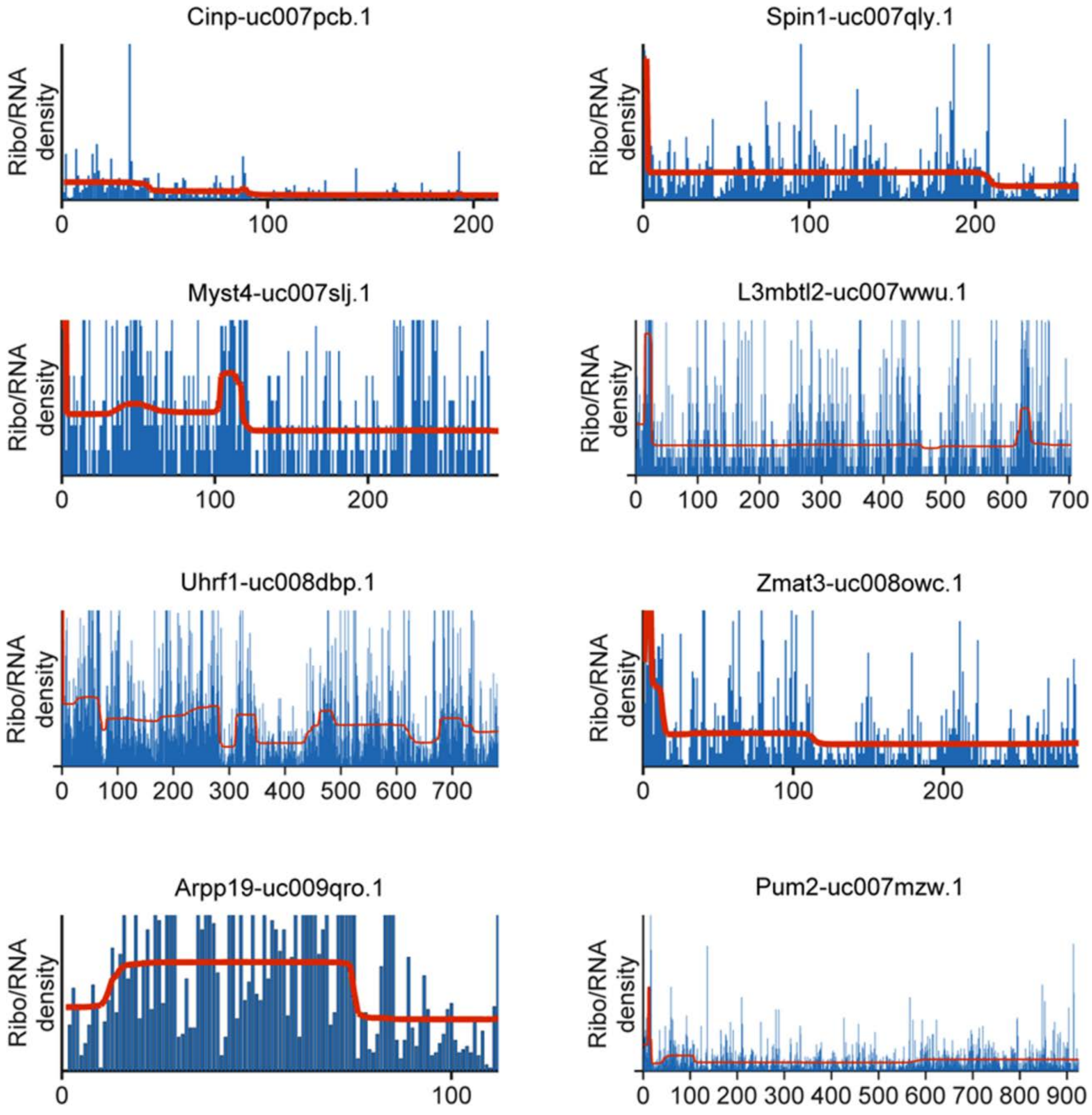

Figure S11. Genes with ribosome drop-off. For these genes a drop in ribosome density was found that could not be explained by alternative termination. As most of the drop of density were preceded by a particularly high density signal, we suggest that ribosome drop-off after ribosome stalling could be underlying cause.

Figure S12

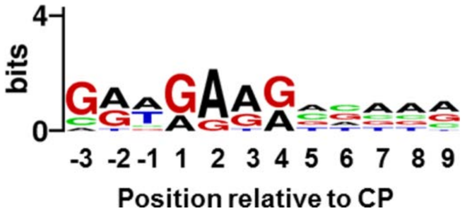

Figure S12. A sequence logo showing the most conserved bases around the point of ribosome drop-off of the genes in Figure S11.

Figure S13

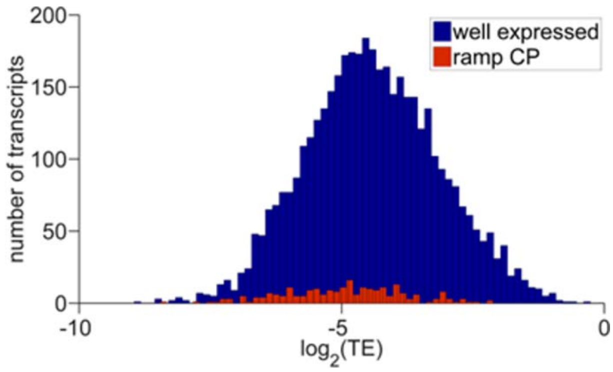

Figure S13. Translational efficiency of well-expressed transcripts and ramp transcripts. The difference between the groups was not statistically significant ($p = 0.238$, t-test).

Figure S14

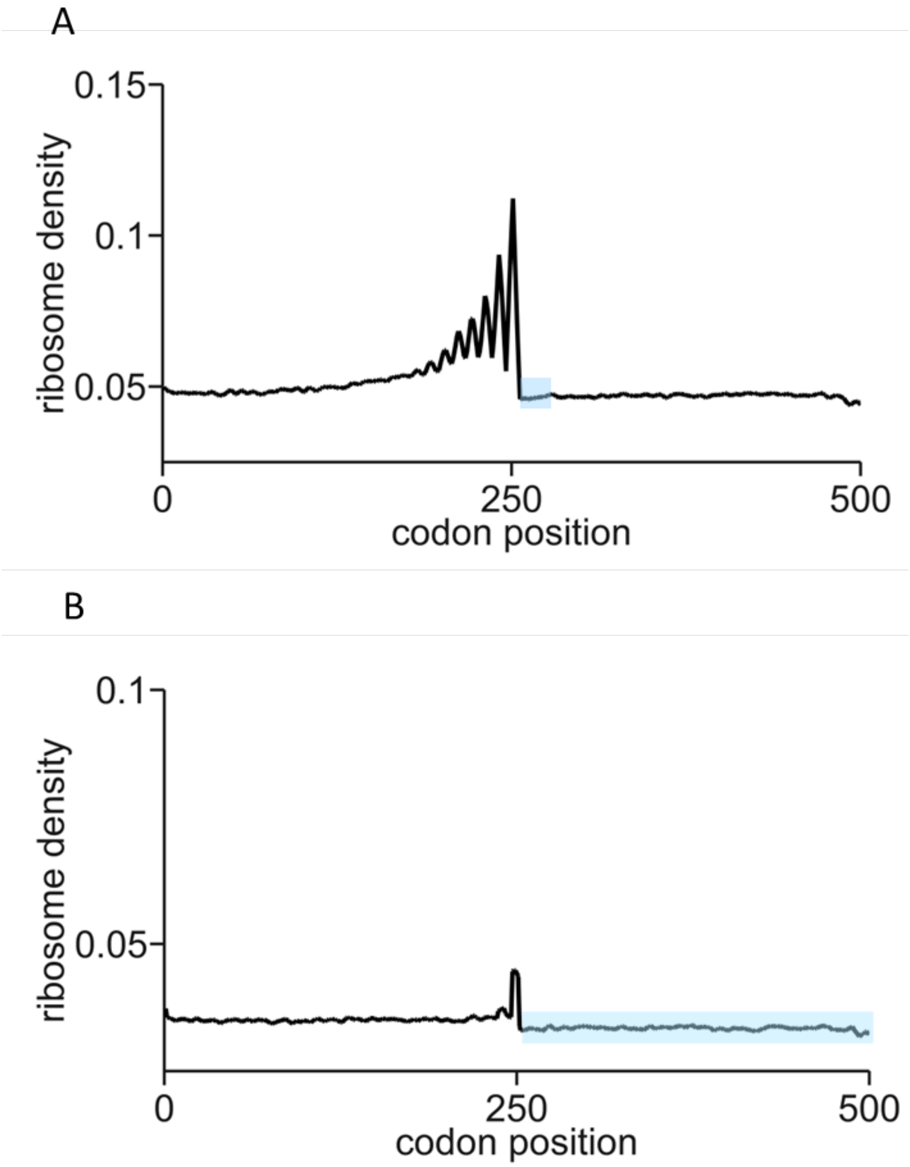

Figure S14. TASEP simulations of translational regulation. Ribosome density along the mRNA for A) a stretch of 5 slow codons ($k_I = 0.08, k_{Ei} = 1, k_{Eslow} = 0.4, k_T = 1$) and B) 0.95 probability of ribosome drop-off at single slow codon ($k_I = 0.05, k_{Ei} = 1, k_{Eslow} = 0.4, k_T = 1$). Blue rectangle shows the length along the transcript after the slow codons, in which ribosome density is decreased. For all simulations $N = 500, L = 10, n = 100000$ (number of samples taken from the simulation).

Table S1

Regulation type	Parameter value
Slow initiation	k_I = 0.5
Slow termination	k_T = 0.2*
Slow elongation 5’UTR	k_{E6} =0.5*
Slow elongation CDS	k_{E251} =0.2*
Slow elongation 3’UTR	k_{E496} =0.2*
Ramp	k_{E1-30} =0.95
Alternative initiation	k_{Alt} =0.01
Ribosome drop-off	k_d =0.01

Table S1. CP detection of translational regulation for computationally generated ribosome profiles. Parameters that lead to CP detection at confidence level above 95 % are given. Other translation parameters used in the simulations are reported in Figure 1. In all cases except when ribosome stalling occured ((*) - $w_0 = 0.2, p_0 = 0.1$), the change point algorithm parameters used were $w_0 = 0.02, p_0 = 0.001$.
